# Supplementary figures and images for: Cryo-EM structures and binding of mouse and human ACE2 to SARS-CoV-2 variants of concern indicate that mutations enabling immune escape could expand host range
Source: PLoS Pathog. 2023 Apr 5;19(4):e1011206. doi: 10.1371/journal.ppat.1011206 (PMC10109501; doi:10.1371/journal.ppat.1011206)

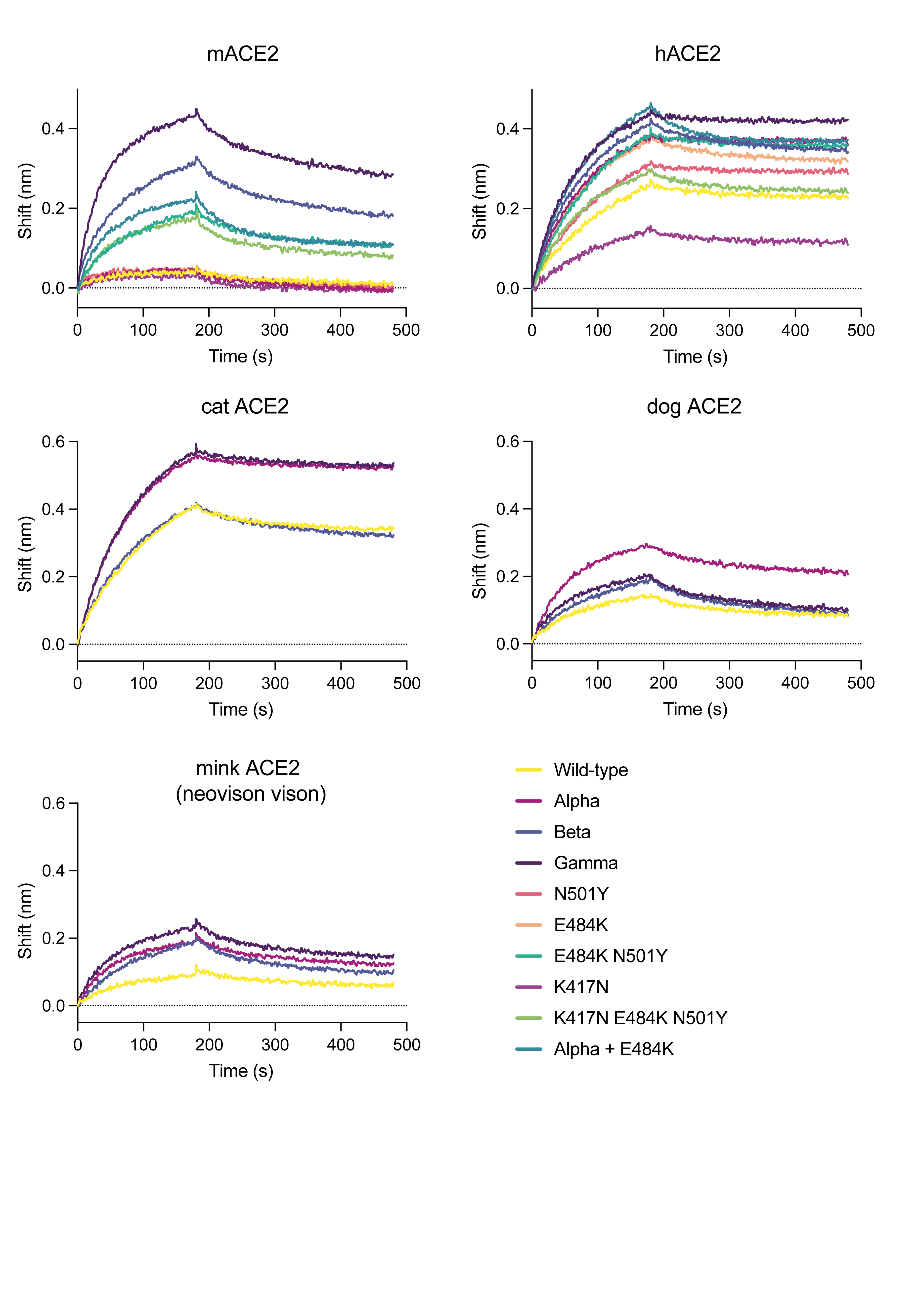

Supplement: S1 Fig — (TIF) [file ppat.1011206.s001.tif]

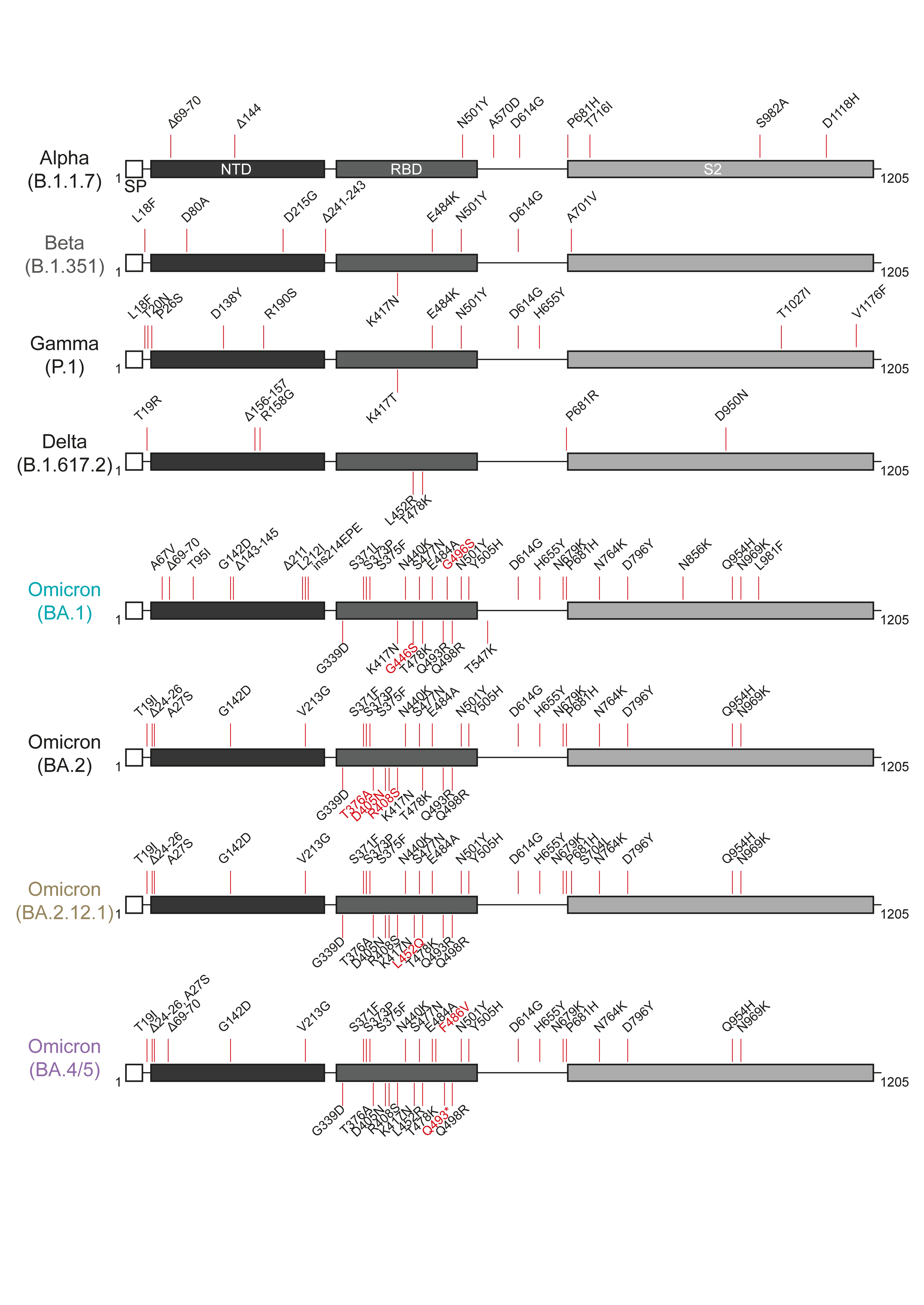

Supplement: S2 Fig — Mutations are shown as red lines and labelled for each variant. Specific domains are highlighted: signal peptide (SP), N- terminal domain (NTD), receptor binding domain (RBD), S1 and S2 domain. (TIF) [file ppat.1011206.s002.tif]

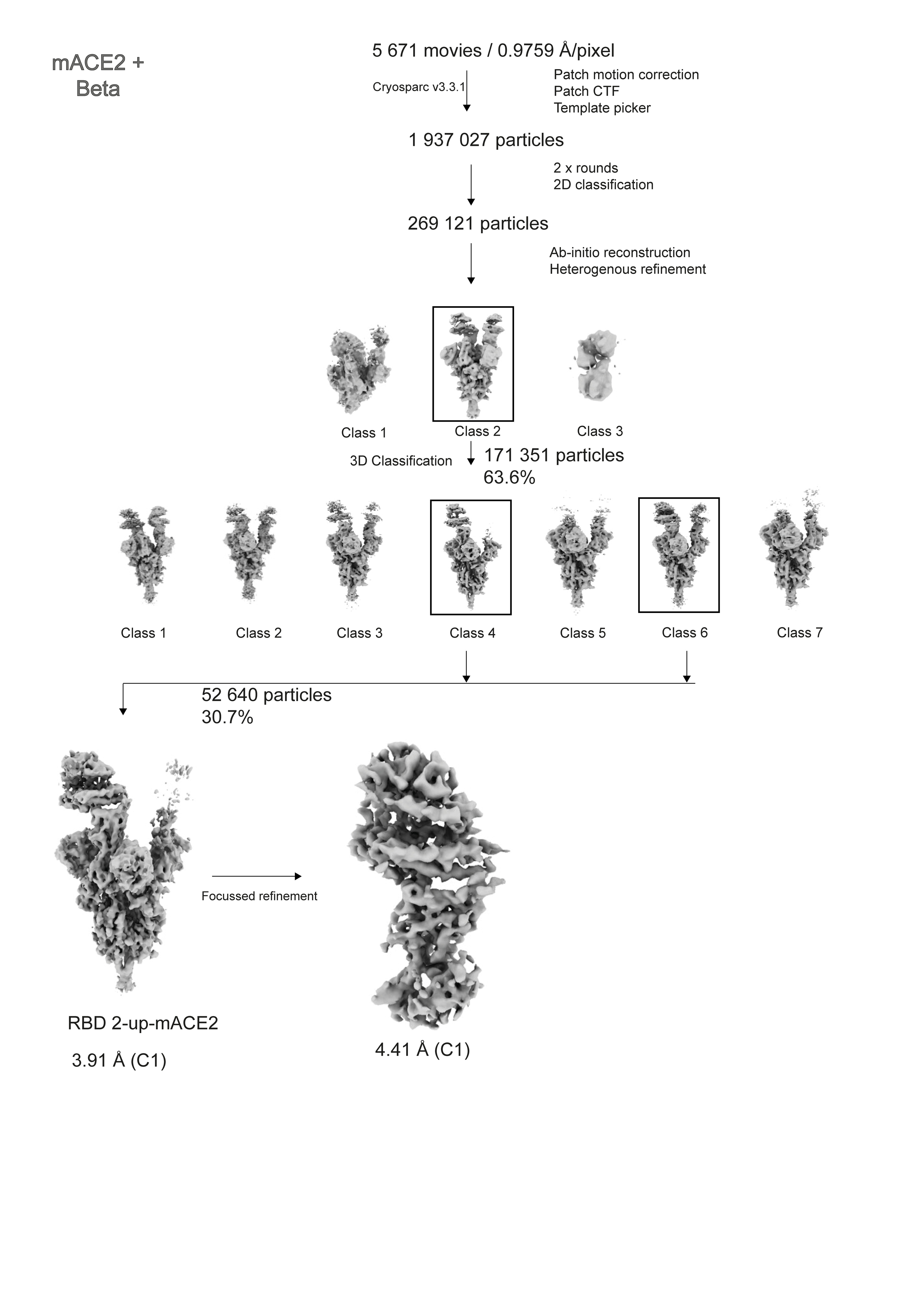

Supplement: S3 Fig — (TIF) [file ppat.1011206.s003.tif]

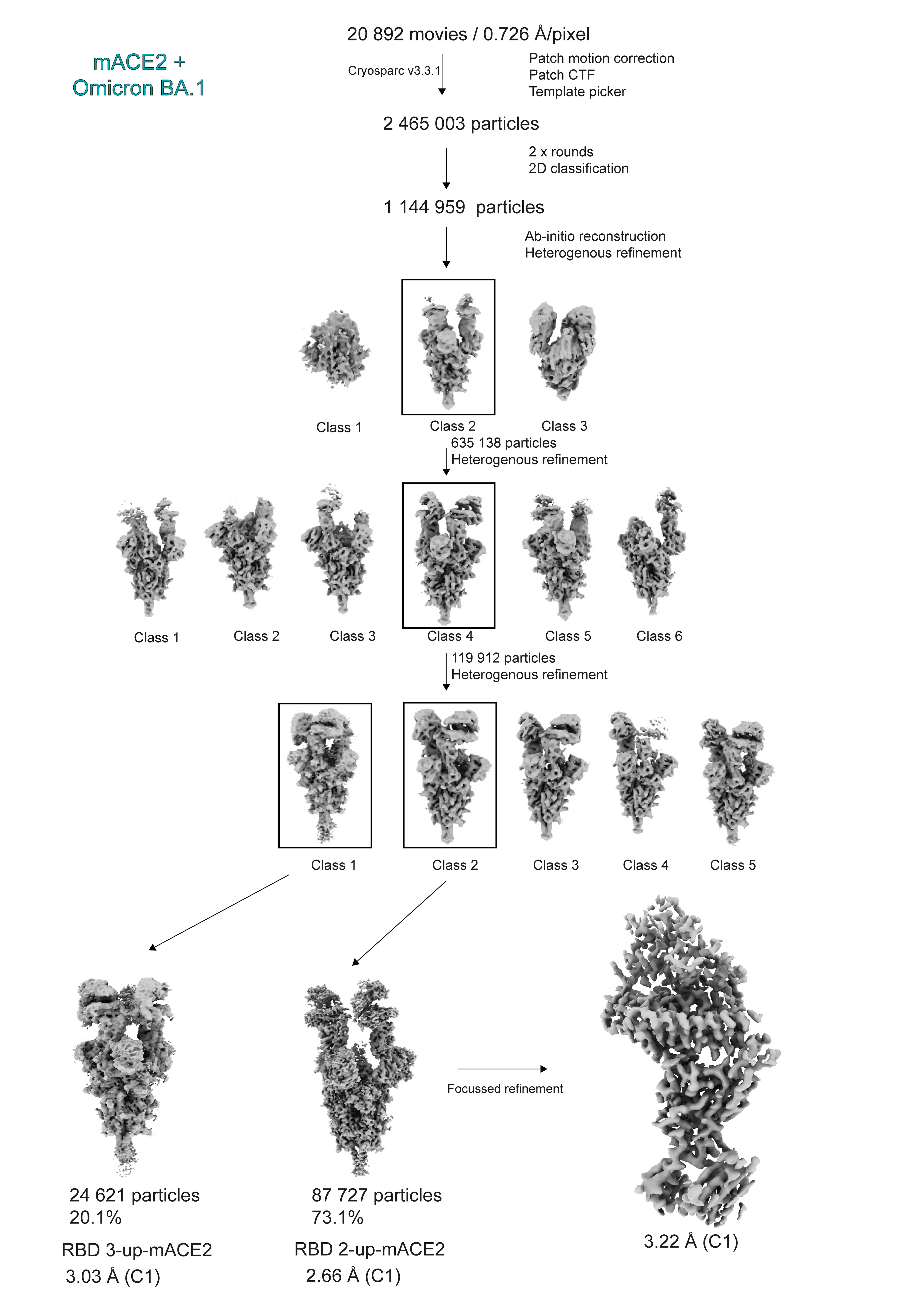

Supplement: S4 Fig — (TIF) [file ppat.1011206.s004.tif]

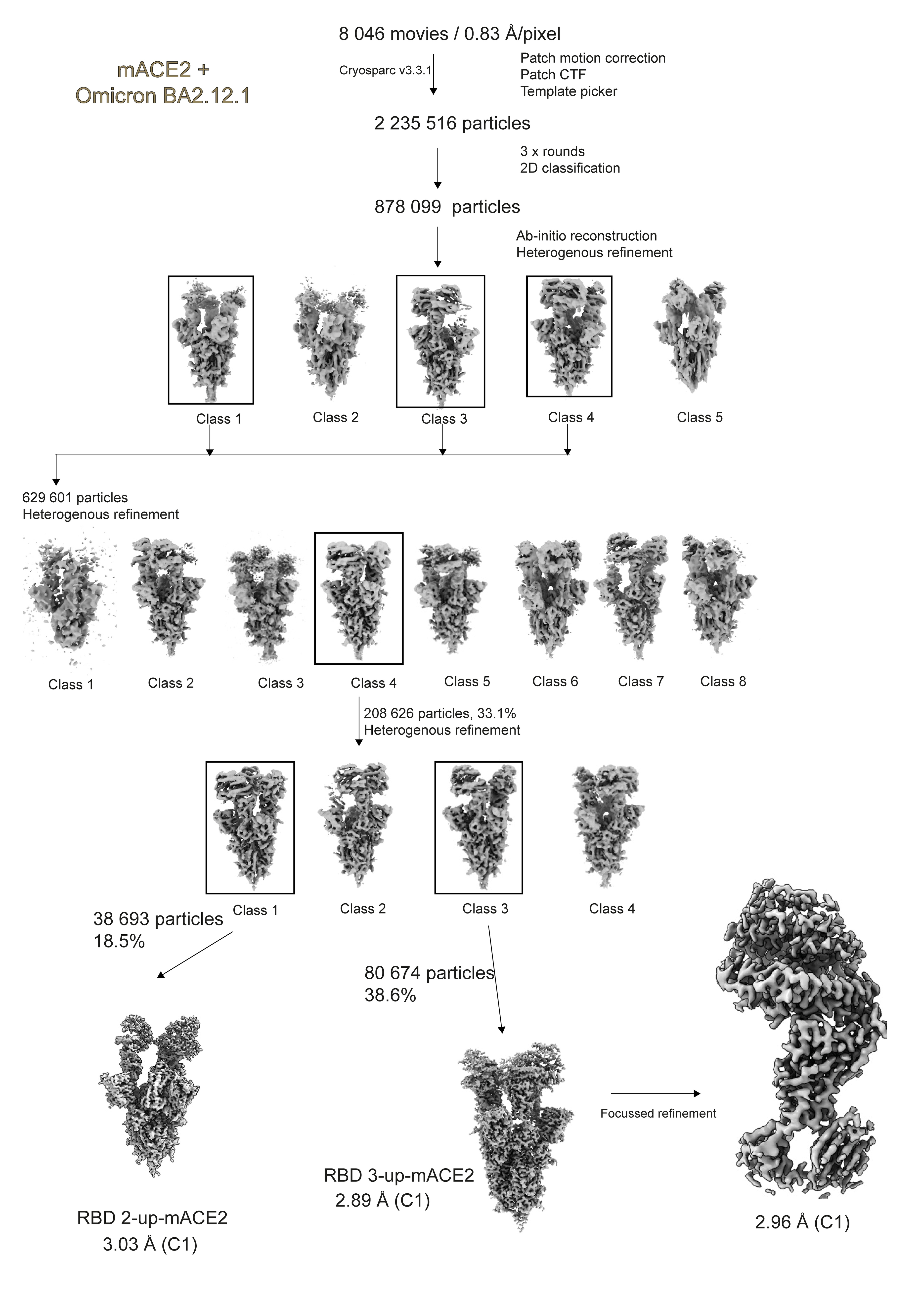

Supplement: S5 Fig — (TIF) [file ppat.1011206.s005.tif]

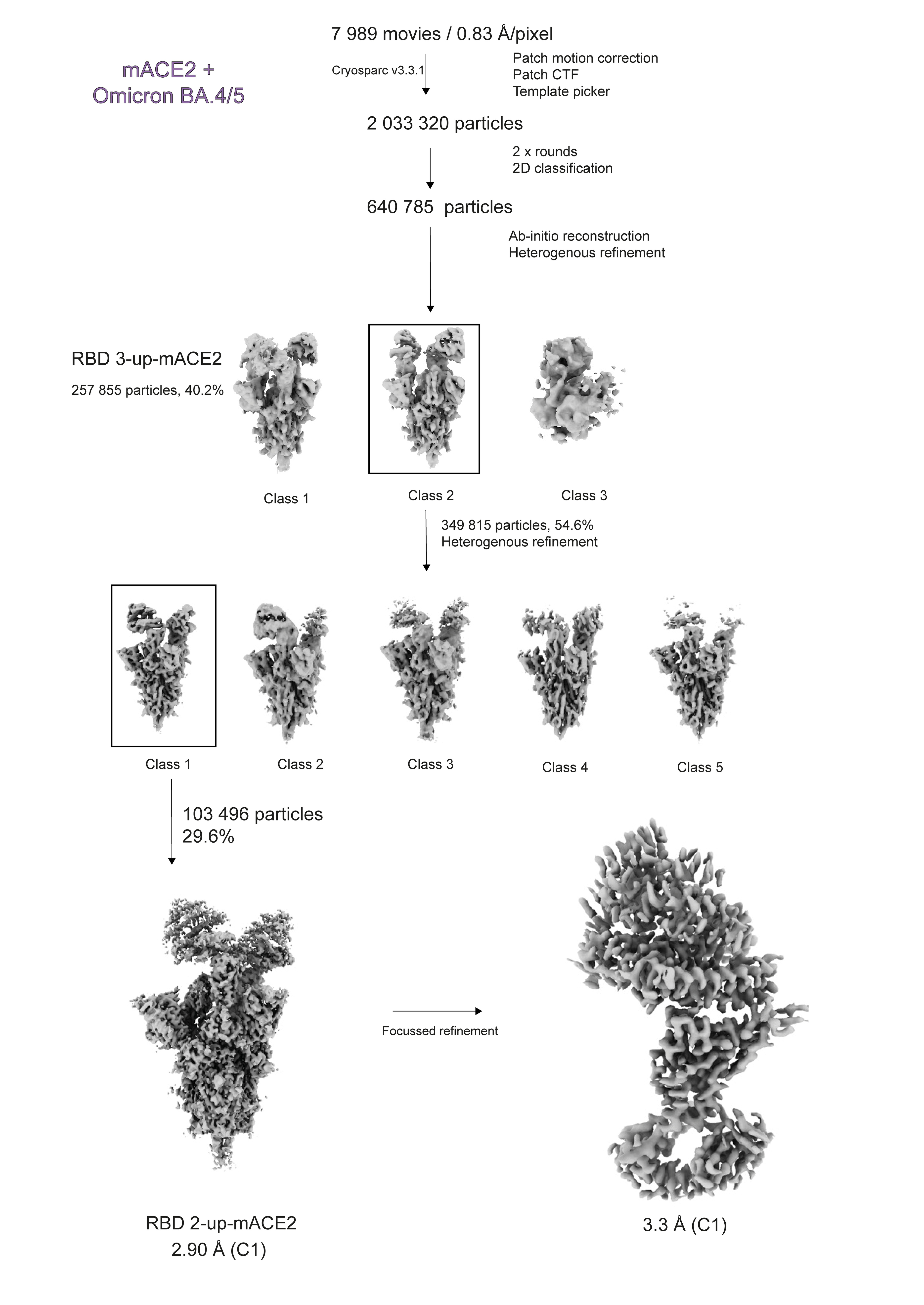

Supplement: S6 Fig — (TIF) [file ppat.1011206.s006.tif]

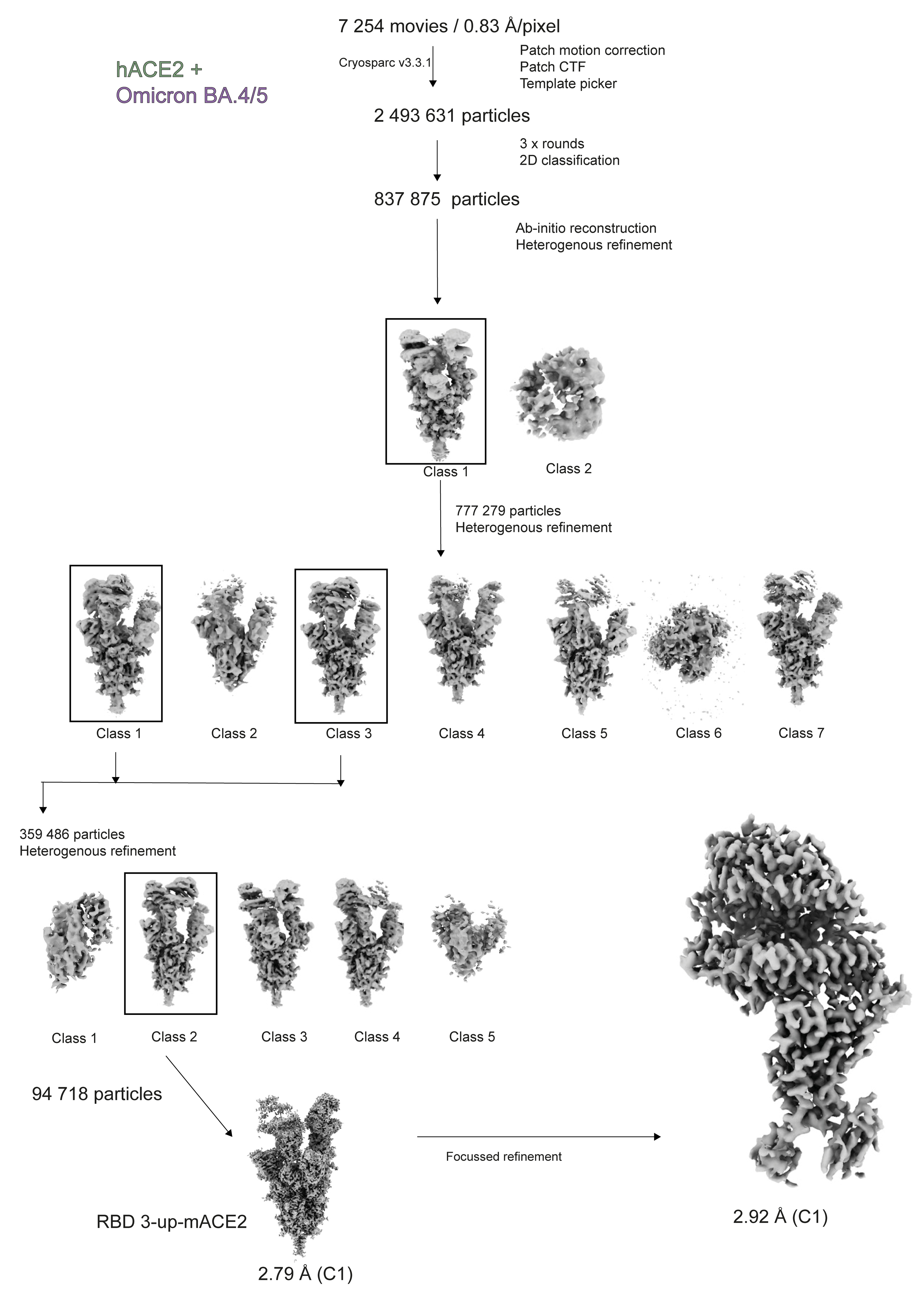

Supplement: S7 Fig — (TIF) [file ppat.1011206.s007.tif]

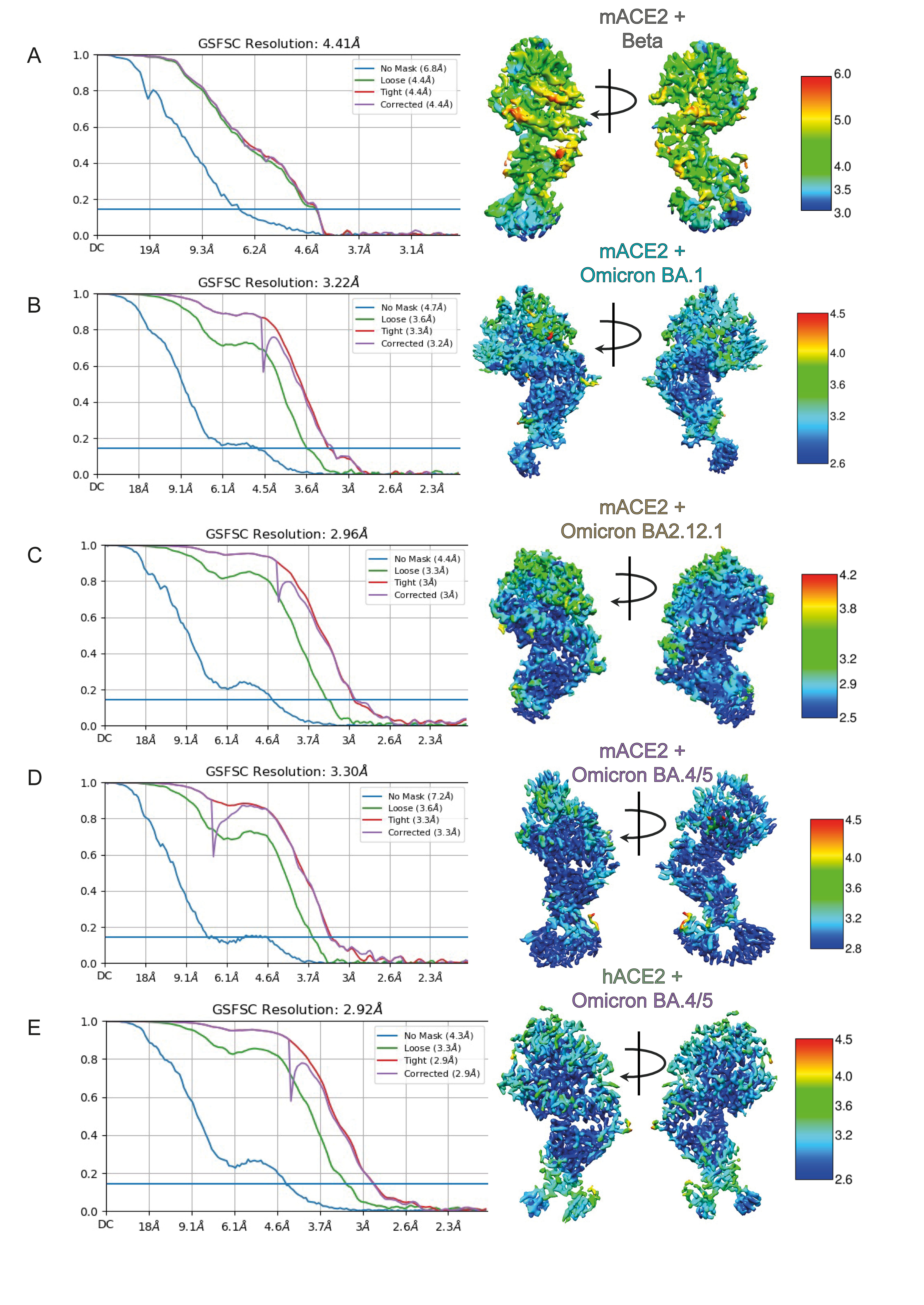

Supplement: S8 Fig — (TIF) [file ppat.1011206.s008.tif]

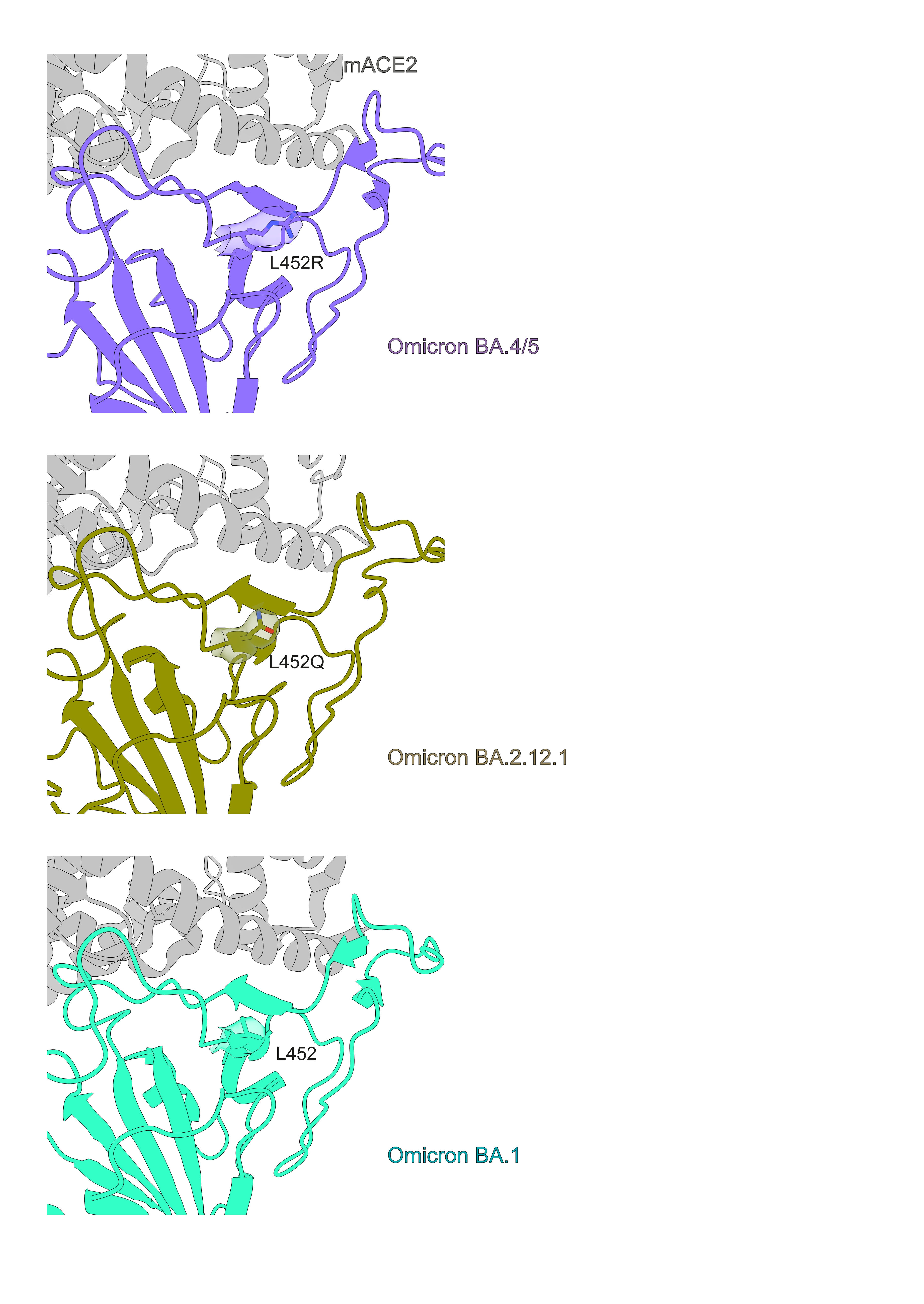

Supplement: S9 Fig — (TIF) [file ppat.1011206.s009.tif]

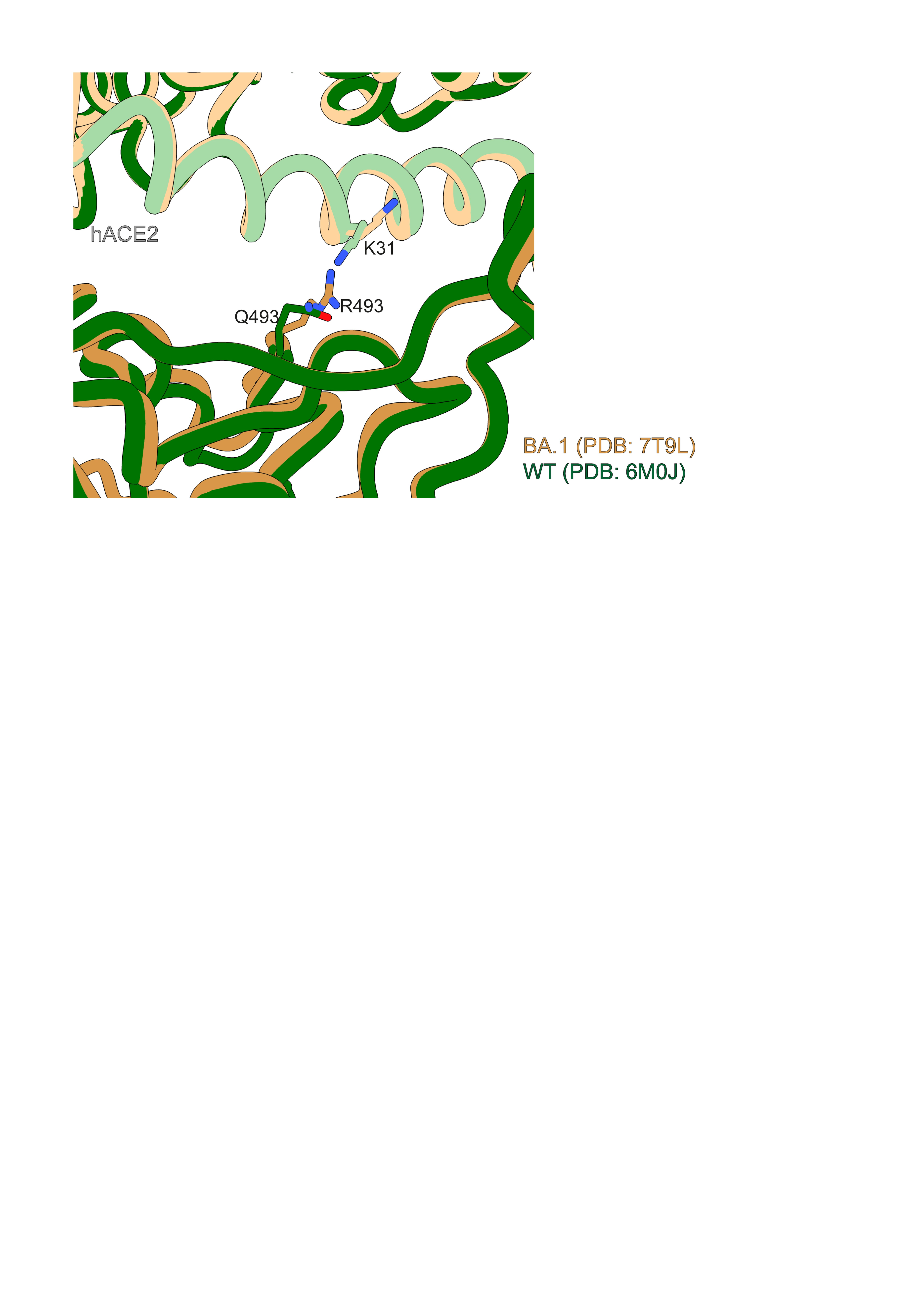

Supplement: S10 Fig — (TIF) [file ppat.1011206.s010.tif]
