# Supplementary material for: Cryo-EM structures and binding of mouse and human ACE2 to SARS-CoV-2 variants of concern indicate that mutations enabling immune escape could expand host range
Source: PLoS Pathog. 2023 Apr 5;19(4):e1011206. doi: 10.1371/journal.ppat.1011206 (PMC10109501; doi:10.1371/journal.ppat.1011206)
Supplement: S1 Table — (DOCX) [file ppat.1011206.s011.docx]

| Data collection and processing | Omicron BA4/5-human ACE2 (EMDB-15588)  (PDB 8AQS) | Beta-mouse ACE2 (EMDB-15589)  (PDB 8AQT) | Omicron BA1-mouse ACE2 (EMDB-15590)  (PDB 8AQU) | Omicron BA2.12.1-mouse ACE2 (EMDB-15591)  (PDB 8AQV) | Omicron BA4/5-mouse ACE2 (EMDB-15592)  (PDB 8AQW) |
| --- | --- | --- | --- | --- | --- |
| Magnification | 96kx | 150kx | 165kx | 96kx | 96kx |
| Voltage (kV) | 300 | 200 | 300 | 300 | 300 |
| Microscope | TFS Titan G4 | TFS Talos Arctica | TFS Titan G4 | TFS Titan G4 | TFS Titan G4 |
| Electron exposure (e–/Å^2^) | 60 | 40 | 60 | 60 | 60 |
| Defocus range (-μm) | -0.8-2.5 | -0.8-2.5 | -0.7-2.0 | -0.7-2.4 | -0.8-2.4 |
| Pixel size (Å) | 0.83 | 0.9759 | 0.726 | 0.83 | 0.83 |
| Symmetry imposed | C1 | C1 | C1 | C1 | C1 |
| Initial particle images (no.) | 837 875 | 269 121 | 1 144 959 | 878 099 | 640 795 |
| Final particle images (no.) | 94 718 | 52 640 | 87 727 | 80 674 | 103 496 |
| Map resolution (Å) | 2.92 | 4.41 | 3.22 | 2.96 | 3.3 |
| FSC threshold | 0.143 | 0.143 | 0.143 | 0.143 | 0.143 |
|  |  |  |  |  |  |
| **Refinement** |  |  |  |  |  |
| Initial model used (PDB code) | n/a | 7QO7, 7FDK | 7QO7, 7FDG | n/a | n/a |
| Map sharpening B factor (Å2) | -68.4 | -135.8 | -78.8 | -63.1 | -46.6 |
| Model composition  Non-hydrogen atoms  Protein residues  Water  Ligands | 6493  788  1  NAG:6 ZN:1 | 6321  777  0  NAG:3 | 6340  777  0  NAG:3 | 6448  789  0  NAG:3 | 6458  789  0  NAG:3 |
| *B* factors (Å^2^) (lowest/highest/mean)  Protein    Ligands  Water | 21.76/204.34/80.80  82.05/134.60/103.02  6.58/6.58/6.58 | 70.88/351.28/158.61  134.44/259.41/206.11  --- | 11.478/220.77/92.89  65.22/118.96/92.25  --- | 17.0/194.06/83.54  54.59/130.61/88.56  --- | 27.46/164.34/75.76  71.27/145.17/101.80  --- |
| R.m.s. deviations  Bond lengths (Å)  Bond angles (°) | 0.002 (0)  0.522 (1) | 0.003 (0)  0.652 (4) | 0.002 (0)  0.606 (5) | 0.003 (1)  0.701 (12) | 0.002 (0)  0.582 (3) |
| Validation  MolProbity score  Clash score  Poor rotamers (%) | 1.39  4.10  0.00 | 1.87  9.08  0.15 | 1.95  8.47  0.15 | 1.61  4.53  0.00 | 1.76  6.12  0.15 |
| Ramachandran plot  Favored (%)  Allowed (%)  Disallowed (%) | 96.81  2.93  0.26 | 94.31  5.56  0.13 | 91.72  8.28  0.00 | 94.39  4.97  0.64 | 93.63  6.11  0.25 |

**S1 Table: Cryo-EM data collection, refinement, and validation statistics**
