# Supplementary material for: Cryo-EM structures and binding of mouse and human ACE2 to SARS-CoV-2 variants of concern indicate that mutations enabling immune escape could expand host range
Source: PLoS Pathog. 2023 Apr 5;19(4):e1011206. doi: 10.1371/journal.ppat.1011206 (PMC10109501; doi:10.1371/journal.ppat.1011206)
Supplement: Attachment — Submitted filename: Reviews-round-1-comments-KL1.docx [file ppat.1011206.s013.docx]

**Point-by-point discussion of reviewer reports and changes**

**We would like to thank the Editor and Reviewers #1-3 for their valuable input and comments. We hope our comments below (in blue) and changes to the manuscript satisfy your concerns and improves the manuscript as a whole. Below is a point-by-point response to your comments and a detailed description of the revisions made in response to their suggestions. We provide a copy of the revised manuscript with all the changes tracked.**

------

This is an important structural study on SARS-CoV-2 spike mutations, species tropism and immune evasion. In general, all three reviewers were positive about the manuscript and did not find any major faults with the experiments or data provided. While there are several points to be addressed in the writing, reviewer 1 was primarily concerned with some of the discussion on species tropism and evolution and reviewer 2 had several structural questions regarding trimeric spike conformation. Reviewer 3 noted that this study does not validate the results with functional assays in the form of cell culture or mouse model experiments. The authors should adjust the manuscript with additional context and detail as necessary, as well as provide a thorough point-by-point rebuttal.

Reviewer Comments (if any, and for reference):

Reviewer's Responses to Questions

**Part I - Summary**
Please use this section to discuss strengths/weaknesses of study, novelty/significance, general execution and scholarship.

Reviewer #1: Lau and colleagues present datasets across a panel of relevant SARS-CoV-2 variants for binding affinity to mouse ACE2 and atomic structures of the interactions. As viral surveillance efforts continue to probe for other animals that contract (or potentially propagate) SARS-CoV-2, it is important to have these complementary biochemical studies to understand the future potential of other spillback pathways. Overall, the datasets are useful. I’m not ordinarily so nitpicky, but I found myself noting a lot of small aspects of writing and scholarship that could be improved, but these are minor comments and I do not have any major critiques on the study details or the datasets that are presented in the manuscript.

Reviewer #2: Ni et al. analyzed the structures of SARS-CoV-2 VOC mutants bound to mouse or human ACE2, and measured the corresponding dissociation constants. The authors claimed that mutations promoting immune escape could also help SARS-CoV-2 achieve cross-species transmission. Note that previous studies have proposed the possibility of a mouse origin of Omicron based on computational analyses; this paper provided critical experimental data to improve our understanding of the evolution of Omicron and therefore would attract a broad group of readers interested in the evolution of SARS-CoV-2, especially the Omicron variant. I found the paper generally easy to follow and would be happy to recommend its publication in PLoS Pathogens after addressing the following questions.

Reviewer #3: This manuscript address the potential concerns of the spillover of SARS-CoV-2 to mouse to establish a new reservoir using the structural investigation. The results give comprehensive and detailed mechanisms for the enhanced binding capacity of the VOCs to mouse ACE2. The main weakness is the lack of confirmation using the functional assays such as cell infectivity of mouse model infections.

**Part II – Major Issues: Key Experiments Required for Acceptance**
Please use this section to detail the key new experiments or modifications of existing experiments that should be absolutely required to validate study conclusions.

Generally, there should be no more than 3 such required experiments or major modifications for a "Major Revision" recommendation. If more than 3 experiments are necessary to validate the study conclusions, then you are encouraged to recommend "Reject".

Reviewer #1: none

Reviewer #2: (No Response)

Reviewer #3: The cell infectivity or mouse model assays could be used to verify the conclusions.

**Thank you Reviewer #3 for this input. As we have stated in our introduction, our goal for this study was to better characterize the structural details of variant of concern SARS-CoV-2 Spikes binding mouse ACE2 by cryo-electron microscopy. This was due to the early data from the Alpha, Beta, Gamma VOC era that showed that indeed those variants can infect mice. When we decided to characterize further VOCs such as Omicron, several reports already tested these variants in a mouse model.**

**We hope that together with cell based in vitro and mouse model work done by other groups, our structural contribution would further our understanding of species tropism. We will include in the manuscript in the discussion to make it clear that our data has limitations if taken alone, but it builds upon the mouse model work done by other groups and are beyond the scope of our aim for this manuscript.**

**We have added the following standalone paragraph to the discussion (lines 268-274):**

**“Our structural study of human and mouse ACE2 bound to variant Spikes is not without limitations. The ability to bind mACE2 does not necessarily imply that it is able to infect and replicate in mouse.[69] However, numerous *in vivo* mouse studies with early-pandemic, Omicron and mouse-adapted variants demonstrate that these variants are able to infect and cause disease[35,44,45,57,70] In particular, our cryo-EM models shed light on the structural basis of mACE2 binding to these variants. Importantly, our data complements previous studies that also identified mutations that enable mACE2 binding, such as N501Y.”**

**Part III – Minor Issues: Editorial and Data Presentation Modifications**
Please use this section for editorial suggestions as well as relatively minor modifications of existing data that would enhance clarity.

Reviewer #1: Line 21-22 – I don’t find the argument that mice may act as reservoirs for SARS-CoV-2 compelling. SARS-CoV-2 doesn’t need a reservoir, it’s an endemic human virus.

Lines 39-41 describe some uncertainty about ultimate reservoir host of these viruses, but I don’t think there’s any doubt that the natural reservoir is Rhinolophus bats (as opposed to “some evidence” suggested in line 41).

**Thank you for the comments in regards to the broadness of the idea of mice as being a reservoir. We do agree that the virus itself is entrenched and endemic.**

**We have made the suggested changes to lines 20-21, replacing it with:**

**“There is significant interest in describing how the virus interacts with mice as they are well adapted to human environments, are used widely as infection models and can be infected.”**

**We have also clarified the lines 29-41 (now lines 56-58) referring to the natural hosts and made it clear that it is accepted within the scientific community that the hosts are bats.**

**“Both viruses are of animal origin and their intermediate hosts prior to the jump to humans have not been precisely identified, however, it is accepted that like the SARS-CoV virus, the original host of the SARS-CoV-2 virus are most likely Rhinophilus bats .[11–14]”**

Citation 16 is likely meant to be a different Walls et al. paper (Cell 2020)?

**Thank you yes this has been corrected, we wanted to refer both to the Walls 2016 (SARS-CoV) and 2020 (SARS-CoV-2) papers.**

One potentially helpful part of context outlined in the introduction (e.g. around line 52), is that many of the spike residues that are dominant targets of neutralizing immunity are also those that influence ACE2 binding specificity and therefore potentially host tropism. Therefore, turnover of amino acids at these positions due to antigenic selection in humans could have the secondary effect of influencing potential host range. This logic is implicit in what I believe the authors are saying, but it could be laid out more specifically in the introduction.

**We thank the reviewer for this valuable comment and expanded upon this as indeed that is the point we are making.**

**We have added this to lines 71-73:**

**“Significantly, many sites of mutations in VOC are located on the receptor binding domain (RBD) that engages ACE2. Mutations that endow the ability for the Spike protein to evade antibodies in humans may also play an additional role by influencing ACE2 binding specificity and host range.”**

Nomenclature and being consistent – e.g. just between lines 66 and 68, BA4/5 and BA 4/5 (with and without space) is used. I believe the correct nomenclature for the Omicron variants has a period in it (e.g., BA.4/5), and I usually see the Greek letters (e.g. Beta) capitalized

**Thank you to this reviewer for noticing this. We have corrected this error in nomenclature here and made it consistent with the correct nomenclature used in the other parts of the paper**

Line 118: “required” implies that no other route toward mACE2 binding is possible in the absence of N501Y, which disagrees with other publications that show other ways of acquiring mACE2 binding independent of N501Y (e.g., PMID 32854108, 35114688). Just because naturally evolved SARS-CoV-2 variants to date have acquired mACE2 binding via N501Y doesn’t mean that mutation is “required” in a global sense.

**Thank you reviewer for suggesting this. We agree that it is only a mutation that we have observed in naturally occurring VOCs and that this does not preclude other evolutionary paths with different mutations that would enable binding to mACE2.**

**We have modified the line 150-151 to make it clear it is one of the residues that enable binding but not absolutely required:**

**“This mutation is located within the RBD and is one residue that has been identified to enable higher affinity interactions with the mACE2 receptor.[4,57,58]”**

Line 134: in e.g. BA.1, 15 of 34 mutations being in the RBD is precisely not a “majority”

**We have added more precision by changing line 165-168 to include:**

**“These variants contain up to 34 mutations scattered across the entire Spike protein, with the 15 of those concentrated on the RBD, including N501Y and E484A (S2 Fig). In particular, some Omicron RBD mutations (K417N, G446S(BA.1), E484A, F486S (BA.4/5) Q493R, G496S(BA.1), Q498R (BA.1, BA.2, BA.2.12.1) and N501Y) have been shown to be associated with immune evasion, with a loss of antibody binding or higher affinity ACE2 binding.[31,59–62]”**

Line 172: “Y453 of mACE2” – Y453 is a spike residue, not a mACE2 residue

**Thank you for catching this error, we have updated this on lines 202-204 and made the sentence clearer:**

**“Surprisingly, compared to BA.1, R493 of BA.2.12.1 forms an alternative hydrogen bonding network with mACE2 residues N31 and E35, instead of Q34, that now forms a new hydrogen bond with Y453 of BA.2.12.1 suggesting a plasticity in its interactions (Fig 4B).”**

Line 183: Why point to molecular modeling for the claim that F486V is affinity-decreasing when it has been shown experimentally? (e.g., <https://doi.org/10.1371/journal.ppat.1010951>).

**Thank you for bringing this to our attention of experimental results also illustrating the effects of this mutation. We have modified this in line 215-217 and added the appropriate references:**

**“It has been predicted by modelling and then demonstrated by deep mutation scans of the RBD that the F486V mutation reduces the affinity of the RBD for hACE2 and before the appearance of the BA.4/5 subvariant, mutation at this site was observed at very low rates.[64,65]”**

Line 220: “… N501Y … also increases binding to mouse-adapted strains of SARS-CoV-2” – some sort of grammatical/logical error here (I think it should be, “also increases binding to mouse ACE2 (and other potential host species) in mouse-adapted strains of SARS-CoV-2” or something to that effect

**We thank the reviewer for noticing this error. We have modified lines 253-254 to now read:**

**“This structural observation is consistent with literature reports that reported Spike mutations N501Y, Q493H and K417N increases binding to ACE2 from mice and other species.[3,4,68]”**

Throughout, over-reliance on ACE2 binding = potential reservoir. Being a reservoir is about more than receptor binding, it depends on ecology and animal population dynamics and viral restriction versus permissivity and transmission and so many other factors. Language therefore needs to be tightened up in this regard, e.g. lines 21 In contrast, an appropriately agnostic statement as a good example is line 237-238.

**We agree that the use of the word reservoir is quite liberal and although it may be the conclusion of some people that mice or other animals may become reservoirs (even as the virus is endemic in humans), we agree that is not the claim we are trying to make. We have removed the reservoir language throughout, and emphasized more on the ability for variants to bind mACE2 and not to speculate if that would lead to any reservoirs or not. The only exception is the case where we refer to specific reports or hypothesis of the mouse-origins of Omicron.**

**Of note, Line 21 now reads:**

**“There is significant interest in describing how the virus interacts with mice as they are well adapted to human environments, are used widely as infection models and can be infected.[2]”**

**Line 74-75 now reads:**

**“The availability of 3D structures of variant RBDs interacting with the receptor is crucial for understanding how they continue to bind not only human ACE2 but potentially those of other animals.”**

**Line 79-80 has been changed to:**

**“To better inform the relationship between these variants and potential species tropism, there is a need to examine the structural details between animal ACE2 receptors and the variant Spike proteins.”**

**Line 231-233 has been changed to:**

**“We report here that since the appearance of the first variant of concern Alpha, the SARS-CoV-2 Spike glycoprotein has serendipitously gained the ability to also bind the mouse ACE2 receptor potentially broadening its host range.[44–47]”**

**Line 255-258 has been changed to:**

**“We further observe that Spike proteins from Omicron subvariants with significant antibody evasion abilities such as BA.2.12.1 and BA.4/5 still bind to mACE2 demonstrating that the SARS-CoV-2 virus can balance both evasion of the human humoral immune response and its ability to bind to different host species.”**

Reviewer #2: 1. The title includes the phrase “expand host range”, which is also present in Discussion (Lines 241-242). However, the only host other than humans tested in the study is mice. It is probably better to specify the alternative host in this case, unless the authors would like to provide additional data about the ACE2-spike interactions in other host species.

**We initially performed a screen on a limited amount of ACE2 homologs of domesticated/companion mammals (mouse, dog, cat and mink (neovison vison)). We did not include our data for the latter three animals during our initial submission as we focussed principally on Spike variants and mouse binding, due to the surprising observation that with wild-type Spike, it was the only animal ACE2 that we observed that did not bind substantially.**

**We have now included our preliminary data with dog, cat and mink ACE2 performed against wildtype, and the early-Pandemic, Alpha, Beta and Gamma variants, where the arrival of these variants also increased binding affinity to these animal ACE2 receptors.**

**We have added this to lines 95-98:**

**“We performed a BLI binding screen between SARS-CoV-2 variants of concern Alpha, Beta and Gamma against a set of animal ACE2, including mouse ACE2 (mACE2), compared to human ACE2 (hACE2). Interestingly, unlike cat, dog and mink ACE2 that could bind to the wild-type Spike protein, mACE2 only showed a substantial binding signal against the VOC, similar to other reports.[22,42,43] (S1 Fig) We decided to focus on and better characterize mACE2 binding affinities against these Spike VOC and performed full BLI binding assays at three concentrations.”**

2. I have doubts if Fig. 1c was correctly cited in the main text (lines 164-165). The difference in amino acid sequences is provided in Fig. 1c but is not explicitly discussed in the main text.

**Thank you for noticing this error. The correct Figure is S1 Fig, now S2 Fig.**

3. The authors should explain in more detail about the 2-up and 3-up conformation to the broad audience.

**Thank you for this comment. We believe it is best to discuss this alongside comment #5**

**The up and down conformations of the RBD of Spike are dependent on many factors such as the mutational background, pH and temperature. It is necessary for the RBD to be in the up conformation to engage with ACE2, as the binding site is at the tip of the domain that is occluded when closed. The proportion of RBD up and down are down can play a role in the binding as more RBD-up would allow for the Spike protein to bind greater amounts of ACE2 present on a cell surface. However, this is also disadvantageous for the virus as it presents epitopes that are the site of neutralizing antibodies. Thus, it has been shown that certain variants have different proportions of up and down RBD in the “apo” unbound state.**

**We have added the following to the paper at lines 126-131:**

**“The RBD is highly mobile and its transition between the down- to its up- state reveals the ACE2 binding site and allows for it to bind ACE2. The process is dynamic, and the proportion of RBD-down and -up has been shown to be dependent on many factors such as mutations, glycans, pH and temperature.[49–56] During cryo-EM data-processing we observed the Spikes with a mixture of 2-up or 3-up conformation, with each up-RBD having an ACE2 bound (S3-S7 Figs).”**

4. The authors compared their structure to PDB: 6M0J, which if I understood correctly represents interactions of monomers between spike RBD and ACE2. I wonder how the trimeric spike and dimeric ACE2 investigated in this study should result in identical structures as those solved from monomeric protein domains. Is this topic worth some elaborated discussion/explanation during revision?

**Thank you for noticing this detail in our methodology. Indeed we used dimeric-ACE2 (in the form of an Fc-fusion linked to the extracellular domain of ACE2). For both BLI and cryo-EM experiments, we used this form of ACE2 to better recapitulate how ACE2 typically exists as a dimer in vivo as a membrane protein. Our EM results show only one ACE2 bound to each RBD dimer as previously observed by many groups. As the hinges on Fc-based fusions (as in native antibodies) are flexible the ACE2 domains are free to interact with the RBD, recapitulating the 6M0J was a crystal structure between the isolated ACE2 extracellular domain and the isolated RBD. Just like an x-ray crystal structure, in EM, flexible regions are difficult to be visualized.**

**The high structural similarity between our complexes and 6M0J suggests that eventhough we are using different constructs and techniques, the folding of the domains and how they interact are the same (binding interface) and are comparable**

**We have added to lines 121-123:**

**“Our cryo-EM structures determined with dimeric Fc-ACE2 and a trimerized Spike ectodomain recapitulates the hACE2/wild-type complexes observed in other studies done by x-ray crystallography (PDB 6M0J) or cryo-EM (PDB 7A94), with monomeric ACE2 and RBD or monomeric ACE2 and trimerized Spike, respectively.”**

5. I would suggest the authors introduce more information about the 2-up and 3-up conformations of ACE2. They appeared in lines 101-102 and supplementary figures without sufficient context. And why did only 2-up show up in the mACE2+Beta structure (Fig. S2)?

**Please see our response to comment #3 as this overlaps. Specifically for the Beta structure, we could observe additional 3D classes that may correspond to a 3-up bound class. However, we only selected the 2-up class and its most well resolved bound-RBD to perform local refinement.**

**We have updated lines 130-134 to state that:**

**“During cryo-EM data-processing we observed all the Spikes with a mixture of 2-up or 3-up conformation, with each up-RBD having an ACE2 bound (S3-S7 Figs). To gain structural information at side-chain level resolution of the binding interface, we performed image processing of the cryo-EM data by location-focused refinement on an ACE2-bound RBD that was the most well resolved.”**

6. Figure 3c in the legend should be Figure 3b.

**We have corrected this error**

7. It is not clear how structures in Figure 2c and Figure 3b were aligned. In my view the authors should align mACE2 (or hACE2) for all structures, so that we can see how the Omicron variants structurally varied.

**We have noted in the figure legends more explicitly what was aligned in Figures 2C and Figures 3B. To make it clearer what was superimposed we have added on lines 298 :**

**“Superposition of RBD-mACE2 complexes on to the BA.1-mACE2 complex show mACE2 binds similarly for all variants”**

**and lines 305:**

**“Superposition of BA.4/5 RBD-hACE2 complex with the BA.1 RBD-hACE2 complex (PDB 7T9L).”**

**We also add a new panel to Figure 2D that compares our human and mouse ACE2 bound to BA.4/5 that shows that the binding interface is very similar. The main difference is the relative orientation of the two lobes of ACE2.**

**We have made changes to line 139-142:**

**“The hACE2/BA.4/5 complex is also highly superimposable with that of the crystal structure of the isolated wildtype RBD in complex with monomeric hACE2 (PDB: 6M0J) with an RMSD of 0.9 Å and with movements principally at the ACE2 lobe distal to the interaction site with the RBD (Fig 3B). The hACE2/BA.4/5 complex is very similar to the mACE2/BA.4/5 complex with an overall RMSD of 0.6 Å (Fig 2D).”**

8. Back to the title, the authors did not really show that all these mutations of Omicron variants were associated with immune escape in this study. Probably some indeed contributed to immune escape while others might not. Therefore, I am not sure if it is appropriate to have them in the title.

**We concur that some mutations are not directly involved in immune escape but they directly enable the Spike to acquire immune evasive epistatic mutations ie. Increased ACE2 binding affinity to compensate for loss of affinity elsewhere that allows for escape. (Starr et al., 2022.** [**https://www.science.org/doi/full/10.1126/science.abo7896**](https://www.science.org/doi/full/10.1126/science.abo7896)**)**

**We believe that the title should convey that mutations that enable immune escape can play a role in binding of ACE2 receptors of other hosts. We have modified it slightly to be more precise.**

**Cryo-EM structures and binding of mouse and human ACE2 to SARS-CoV-2 variants of concern indicate that mutations enabling immune escape could expand host range**

Reviewer #3: The Figure 2 and Figure S9 could not totally align. In figure 2, the binding capacity to mACE2 for the VOC is wildtype<beta<alpha<gamma</beta<alpha<gamma

**Thank you for the comment, but we cannot fully interpret this comment. We assume Reviewer 3 is referring to Figure 1 (BLI curves) not Figure 2 (structures), in regards to binding capacity based on the BLI curves.**

**In Figure 1 where we quantitate the binding affinity we see that Alpha, Beta and Gamma are highly similar. As we write in lines 120-121, we note that they are only stronger compared to WT but do not comment on their relative strength.**

**S1 Fig (former S9) was the initial binding screen we performed to screen a panel of animal ACE2 that could bind to either wild-type, Alpha, Beta or Gamma variants. The concentrations used in Fig S1 are 75 nM, between the two lowest concentrations used in Figure 1 between 33-100 nM. As such the curves cannot truly align. The proteins used were also from different batches that may also result in different max binding capacities. As we did not perform a fuller kinetic analysis in S10, as we only used one concentration, we did not quantitate the binding affinities here, but based our interpretation solely on the signal not a fit.**

PLOS authors have the option to publish the peer review history of their article ([what does this mean?](https://journals.plos.org/plospathogens/s/editorial-and-peer-review-process#loc-peer-review-history)). If published, this will include your full peer review and any attached files.


If you choose “no”, your identity will remain anonymous but your review may still be made public.


**Do you want your identity to be public for this peer review?** For information about this choice, including consent withdrawal, please see our [Privacy Policy](https://www.plos.org/privacy-policy).

Reviewer #1: No

Reviewer #2: No

Reviewer #3: No
